# Superficial epithelioma with sebaceous differentiation involving the eyelid: a case report

**DOI:** 10.1186/1752-1947-8-466

**Published:** 2014-12-29

**Authors:** Sultan S Aldrees, Pablo Zoroquiain, Patrick T Logan, Mohammed F Qutub, Natalia Vila, Vasco Bravo-Filho, Conrad C Kavalec, Miguel N Burnier

**Affiliations:** 1 Henry C. Witelson Ocular Pathology Laboratory, McGill University, 3775 Rue University, Room 216, Montreal, Quebec Canada; 2 Department of Ophthalmology, King Saud University, Riyadh, Saudi Arabia; 3 Department of Ophthalmology, McGill University, Montreal, Canada

**Keywords:** Eyelid, Muir–Torre syndrome, Superficial epithelioma with sebaceous differentiation

## Abstract

**Introduction:**

Superficial epithelioma with sebaceous differentiation is a rare benign epithelial neoplasm. It usually involves the head, neck or the back of a middle-age person. To the best of our knowledge, two ocular cases have been reported in the literature.

**Case presentation:**

A 46-year-old man of Italian descent, with a known history of testicular seminoma treated by orchiectomy with chemotherapy and radiotherapy, presented with a tan-colored lesion measuring 4mm in diameter in his right upper lid that had been growing over 10 months. It was clinically diagnosed as papilloma. An excisional biopsy was done. On histological examination, the lesion was a well-circumscribed and sharply demarcated epithelial tumor attached to the overlying epidermis and characterized by plate-like proliferation of basaloid to squamous cells with clusters of mature sebaceous cells and foci of ductal differentiation. After a follow-up period of 5 months, no recurrence of the lesion has been documented.

**Conclusions:**

Superficial epithelioma with sebaceous differentiation is part of the differential diagnoses of eyelid lesions. Arguments in the literature about the correct nomenclature of superficial epithelioma with sebaceous differentiation have resulted in under-diagnosed cases. The benign histological features and the lack of recurrence support its benign nature. Although no clear association has linked superficial epithelioma with sebaceous differentiation with Muir–Torre syndrome, further clinical correlation and close follow up for patients are recommended.

## Introduction

In 1980, superficial epithelioma with sebaceous differentiation (SESD) was first described by Rothko *et al.* as a distinct, rare and benign neoplasm that falls into the larger group of basaloid tumors showing sebaceous differentiation [1]. In their study, SESD affected multiple body areas of a 48-year-old man. The characteristic histological features of these lesions included superficial plate-like proliferation of basaloid cells in the upper dermis with well-defined borders and broad attachments to the overlying epidermis. Keratin-filled cysts and sebocytes of varying maturity in the form of clusters, lobules or single cells are present in some lesions [1–3]. Pigmentation of basal keratinocytes and a host response of superficial infiltrates of lymphocytes was mentioned by subsequent case reports and case series, in addition to the previously described histopathological features [3].

## Case presentation

**Figure 1:**
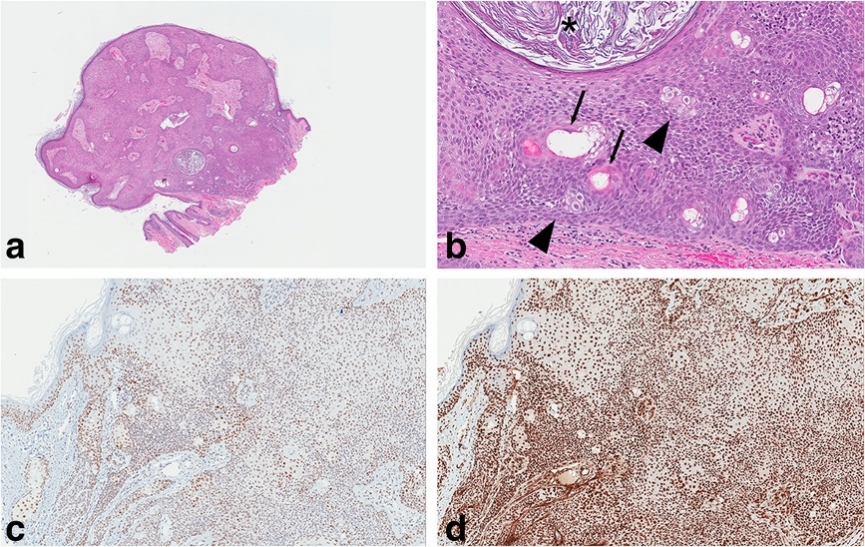
**Histopathological images of superficial epithelioma with sebaceous differentiation. a)** Hematoxylin and eosin stain at low power showing the plate-like proliferation of basaloid cells. **b)** High power showing sebaceous differentiation (arrowheads), keratin-filled cyst (asterisk) and ductal differentiation (arrows). Positive staining of MLH1 **(c)** and MSH2 **(d)** proteins.

A 46-year-old man of Italian descent, with a known history of testicular seminoma that was treated surgically with chemotherapy and radiotherapy, presented to the clinic with a tan-colored, painless papular lesion measuring 4mm in diameter in his right upper lid that grew gradually over 10 months; it was clinically diagnosed as papilloma and an excisional biopsy was performed. He provided no family history of similar eyelid lesions. On histological examination, the lesion was a well-circumscribed and sharply demarcated epithelial tumor, characterized by keratin-filled cysts and superficial basaloid cells with a sheet-like spread in the upper dermis and broad attachments to the overlying epidermis. Aggregates of mature sebaceous cells forming clusters and lobules were arranged replacing basaloid cells in the lower part of the tumor. Furthermore, foci of ductal differentiation were seen, but no mitoses were observed. On immunohistochemical examination, DNA mismatch repair proteins MSH2 and MLH1 were positively expressed in the tumor cells (Figure 1). After a follow-up period of 5 months, no recurrence of the lesion has been documented.

## Discussion

SESD is a rare and benign neoplasm that can affect different body areas [1–9], first described by Rothko *et al*. in 1980 as six cutaneous tumors affecting multiple body areas of a 48-year-old man without any systemic disease or family history [1]. Subsequent case reports and series show that it favors the head, face and trunk of middle-to-old-age individuals regardless of sex [1–10]. On clinical examination, these lesions can grow as rapidly as a few months or slowly over 1 to 3 years and may present as nonspecific papules, nodules or plaques of varying sizes, consistency and color [2, 3, 5]. This broad clinical presentation gives wide differential diagnoses ranging from totally benign lesions, such as sebaceous hyperplasia and seborrheic keratosis, to highly malignant lesions, such as sebaceous carcinoma and basal cell carcinoma [3]. The histopathological features of the lesion also give rise to wide differential diagnoses, mainly that of sebaceous differentiation including, but not limited to, seborrheic keratosis with sebaceous differentiation (SKSD), sebaceous hyperplasia, sebaceous adenoma, sebaceoma, sebaceous carcinoma and basal cell carcinoma with sebaceous differentiation (Table 1) [2]. SESD can be very difficult to distinguish from SKSD histopathologically. However, the presence of squamous like cells arranged in whorls (squamous eddies) that are occasionally found around wide infundibular spaces filled with corneocytes (pseudohorn cysts) is more common in SKSD [3, 4, 11]. Basal cell carcinoma with sebaceous differentiation usually presents with an aggressive behavior and tend to arrange in a lobular pattern rather than the plate-like configuration in SESD [4]. To the best of our knowledge, ocular involvement of SESD has been reported twice in the literature as eyelid lesions in two patients (Table 2) [2, 5].

**Table 1:** **Differential diagnosis of superficial epithelioma with sebaceous differentiation**

No.	Differential diagnoses
1	Seborrheic keratosis with sebaceous differentiation
2	Sebaceous hyperplasia
3	Sebaceous adenoma
4	Tumors of the follicular infundibulum with sebaceous differentiation
5	Sebaceous carcinoma
6	Basal cell carcinoma with sebaceous differentiation

**Table 2:** **Ocular cases of superficial epithelioma with sebaceous differentiation**

Authors	Year	Age	Sex	Location	Clinical description	Recurrence
Friedman *et al.* [5]	1987	57	M	Eyelid	4mm pearly papule	No recurrence with 8-months f/u
Kato and Ueno [2]	1992	38	F	Right upper lid	6mm yellow plaque	No recurrence with 6-months f/u
Our report	2014	46	M	Right upper lid	4mm tan-colored papule	No recurrence with 5-months f/u

*Abbreviations:* *F* Female, *f/u* Follow up, *M* Male.

Currently, the literature is full of different terms describing tumors with foci of sebaceous differentiation. The difficulty in diagnosing these lesions has led to the use of inappropriate terms and, as a consequence, cases of SESD are underdiagnosed cases. Reticulated acanthoma with sebaceous differentiation, SKSD, acanthomatous superficial sebaceous hamartoma, sebocrine adenoma, sebomatricoma and poroma with sebaceous differentiation are all names for SESD in the literature. Despite the different nomenclature, the unifying architecture of different cases of SESD, specifically the broad plate-like outgrowth, favors SESD as the correct description [3, 10, 12]. In addition, different immunohistochemical studies can differentiate this tumor from others [2, 3, 10].

Different reports show a lack of association between SESD and Muir–Torre syndrome (MTS) [1–3, 5, 12, 13]. Although our patient had orchiectomy for a testicular seminoma, the expression of mismatch repair proteins MLH1 and MSH2 was positive, which excludes any microsatellite instability and supports the lack of an association with MTS.

The histogenesis of the tumor is not yet known. Friedman *et al*. [5] speculated that it originates from the pilosebaceous unit. This speculation is supported by Kawachi *et al*. [10] and their immunohistochemical studies, in which they discovered its differentiation toward the sebofollicular epithelium. On the basis of the immaturity of the sebaceous cells in the lesion and the negative staining for epithelial membrane antigen and carbonic anhydrase-2, Kato and Ueno [2] suspected the epidermal pluripotential cells as the origin of SESD and not the pilosebaceous unit.

## Conclusions

SESD is part of the differential diagnoses of eyelid lesions. The difficulty in diagnosing SESD and the arguments in the literature about classifying SESD as a separate entity or as part of other lesions with sebaceous differentiation has resulted in underdiagnosed or mistermed cases. The benign histological features and the lack of recurrence support its benign nature. Although no clear association has linked SESD with MTS, further clinical correlation and close follow up for patients are recommended.

## Consent

Written informed consent was obtained from the patient for publication of this case report and any accompanying images. A copy of the written consent is available for review by the Editor-in-Chief of this journal.

## Abbreviations

MTS: Muir–Torre Syndrome
SESD: Superficial epithelioma with sebaceous differentiation
SKSD: Seborrheic keratosis with sebaceous differentiation.
